# A Missense Mutation in the Alpha-Actinin 1 Gene (*ACTN1*) Is the Cause of Autosomal Dominant Macrothrombocytopenia in a Large French Family

**DOI:** 10.1371/journal.pone.0074728

**Published:** 2013-09-17

**Authors:** Paul Guéguen, Karen Rouault, Jian-Min Chen, Odile Raguénès, Yann Fichou, Elisabeth Hardy, Eric Gobin, Brigitte Pan-petesch, Mathieu Kerbiriou, Pascal Trouvé, Pascale Marcorelles, Jean-francois Abgrall, Cédric Le Maréchal, Claude Férec

**Affiliations:** 1 Institut National de la Santé et de la Recherche Médicale (INSERM), U1078, Brest, France; 2 Faculté de Médecine et des Sciences de la Santé, Université de Bretagne Occidentale (UBO), Brest, France; 3 Laboratoire de Génétique Moléculaire et d’Histocompatibilité, Centre Hospitalier Universitaire (CHU) Brest, Hôpital Morvan, Brest, France; 4 Laboratoire d’Hématologie, Centre Hospitalier Universitaire (CHU) Brest, Hôpital Cavale Blanche, Brest, France; 5 Etablissement Français du sang (EFS) – Bretagne, Brest, France; 6 Service d’Anatomie Pathologique, Centre Hospitalier Universitaire (CHU) Brest, Hôpital Morvan, Brest, France; Auburn University, United States of America

## Abstract

Inherited thrombocytopenia is a heterogeneous group of disorders characterized by a reduced number of blood platelets. Despite the identification of nearly 20 causative genes in the past decade, approximately half of all subjects with inherited thrombocytopenia still remain unexplained in terms of the underlying pathogenic mechanisms. Here we report a six-generation French pedigree with an autosomal dominant mode of inheritance and the identification of its genetic basis. Of the 55 subjects available for analysis, 26 were diagnosed with isolated macrothrombocytopenia. Genome-wide linkage analysis mapped a 10.9 Mb locus to chromosome 14 (14q22) with a LOD score of 7.6. Candidate gene analysis complemented by targeted next-generation sequencing identified a missense mutation (c.137GA; p.Arg46Gln) in the alpha-actinin 1 gene (*ACTN1*) that segregated with macrothrombocytopenia in this large pedigree. The missense mutation occurred within actin-binding domain of alpha-actinin 1, a functionally critical domain that crosslinks actin filaments into bundles. The evaluation of cultured mutation-harboring megakaryocytes by electron microscopy and the immunofluorescence examination of transfected COS-7 cells suggested that the mutation causes disorganization of the cellular cytoplasm. Our study concurred with a recently published whole-exome sequence analysis of six small Japanese families with congenital macrothrombocytopenia, adding *ACTN1* to the growing list of thrombocytopenia genes.

## Introduction

Inherited thrombocytopenia is a very heterogeneous group of disorders characterized by a reduced number of blood platelets that may or may not be associated with a bleeding tendency, which can range from mild to severe [1]. Our understanding of its genetic basis has been greatly improved over the past decade. To date, at least 17 genes and a large chromosomal deletion have been identified as responsible for the different forms of thrombocytopenia [1]. *MYH9* (MIM 160775) is the most frequently affected gene [2,3], followed by other less common ones, such as *FLNA* [4] (MIM 606672), *FLI1* [5] (MIM 193067), *TUBB1* [6] (MIM 612901), *CBFA2* [7] (MIM 151385), *ITGA2B* [8] (MIM 607759), *ITGB3* [9] (MIM 173470), *ANKRD26* [10] (MIM 313900) and *CYCS* [11] (MIM 612004). These findings have allowed refinement of the genotype-phenotype relationship in this condition. For example, the different syndrome forms associated with thrombocytopenia known as May-Hegglin anomaly (MIM 155100), Epstein syndrome (MIM 153650), Fechtner syndrome (MIM 153650) and Sebastien syndrome (MIM 605249) are actually all caused by mutations in the *MYH9* gene and hence represent differently expressed forms of a single genetic disease [12].

One subgroup of inherited thrombocytopenia is characterized by abnormally large or even giant platelets and is thus known as inherited macrothrombocytopenia [1]. Of this subgroup, some thrombocytopenia forms are associated with syndrome features that are suggestive of their underlying causative genes. For example, macrothrombocytopenia associated with leukocyte inclusion, cataracts, nephropathy and/or deafness is often caused by mutations in the *MYH9* gene [2,3] whilst the Gray Platelet Syndrome characterized by the presence of large and “pale” platelets is often caused by mutations in the *NBEAL2* gene [13–15]. There are however very few cases wherein macrothrombocytopenia is the only clinical finding [4,6]. Herein, we describe a large family with autosomal dominant and isolated macrothrombocytopenia and report the identification of its causative mutation by means of positional cloning complemented with next-generation sequencing (NGS).

## Materials and Methods

### Ethics statement

This study was approved by the Hospital’s ethics committee (CPP Ouest VI), and all participants or parents/guardians gave written informed consent.

### The proband with thrombocytopenia

**Figure 1:**
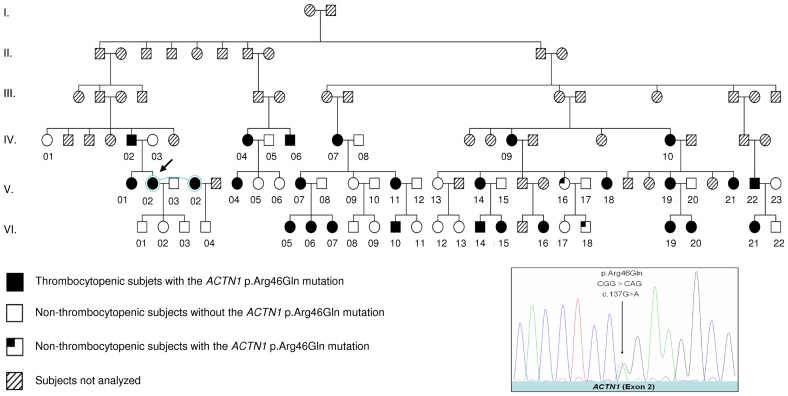
*ACTN1* p.Arg46Gln in the French macrothrombocytopenia pedigree with a model of autosomal dominant inheritance. The arrow indicates the proband. The sequencing electropherogram of the heterozygous *ACTN1* p.Arg46Gln missense mutation is shown in the right-hand bottom box.

The proband is a female of Caucasian origin. She was found to have moderate thrombocytopenia during a laboratory test more than 20 years ago. She was misdiagnosed with immunologic thrombocytopenic purpura at the time because steroid therapy and splenectomy did not improve her platelet count. Her pedigree was constructed through genealogical studies (Figure **1**).

### Platelet count, platelet aggregation study and morphology analysis of bone marrow cells

The platelet count was performed in an automated blood analyzer XE-2100 (Sysmex Corporation, Kobe, Japan). Platelet aggregation was measured on platelet-rich plasma adjusted to 115 G/l, using a Thrombo-aggregometer TA-4V (Sodorel, Heillecourt, France) as described previously [16]. Morphology of the bone marrow cells was examined by optical microscope in according with standard procedures.

### Megakaryocyte *in vitro* culture and electron microscopy

Hematopoietic progenitor cells from bone marrow were harvested after bone marrow aspiration. The cells were cultured at 37°C for 11 days in MegaCult^TM^- *C* *medium* with cytokines (rhThrombopoietin, rhIL-6, rhIL-3) in accordance with the standard procedure, as described previously [17]. Megakaryocyte (MK) cells were then fixed in 1.25% glutaraldehyde in 0.1M phosphate buffer, pH 7.2 (Sorensen buffer), for 30 min at room temperature (RT) after washing the cells with Sorensen buffer. This step was followed by incubation in 1% osmic acid for 30 min and dehydrated in increasing ethanol concentrations (from 50% to absolute ethanol). Before embedding in epoxy araldite resin, the cells were incubated in a propylene oxide solution. The samples were then processed for electron microscopy according to standard procedures, and sections were analyzed using a JEOL JEM-1010 transmission electron microscope.

### Linkage analysis and fine mapping

Genomic DNA was prepared from peripheral blood using standard techniques. All available subjects were typed for the 400 microsatellite markers spread across the 22 autosomes and the X chromosome (average resolution, 9.2 cM; heterozygosity, 0.79) of the ABI PRISM Linkage Mapping Set MD10 (LMS version 2.5, Applera, Foster City, USA), according to the manufacturer’s instructions. Fragment analysis of the amplified products was performed using a 3130xl ABI Genetic Analyzer (Applera) and the GeneMapper software v3.2 (Applera) for data interpretation.

Multipoint parametric linkage analyses were performed with SimWalk v2.91 under the graphical user interface easyLINKAGE v5.08 [18], with the assumption of a dominant mode of inheritance with incomplete penetrance (0.01, 0.95 and 0.95 for the wild-type homozygote, mutant heterozygote and mutant homozygote, respectively). As suggested for extended families, a codominant allele frequency algorithm was used for the analysis; the frequency of the disease allele was set at 0.001. Additional fine mapping was performed in the candidate region using microsatellite markers selected from public databases (data available upon request).

### Candidate gene selection and mutational analysis

Candidate genes within the linkage-mapped locus were selected on the basis of their expression profiles in bone marrow and blood as well as their functional relationship to known thrombocytopenia genes. The top candidate gene, *ACTN1*, was analyzed first; 22 primer pairs were designed to PCR amplify its 22 exons and their immediate flanking sequences (primer sequences and PCR conditions available upon request). The PCR products were purified by ExoSAP-IT (GE Healthcare, Orsay, France) and then sequenced using the ABI PRISM™ BigDye™ Terminator Cycle Sequencing Kit v.1 (Applera).

### Targeted sequencing of the mapped locus by NGS

A custom-made SureSelect oligonucleotide probe library was designed to capture the sequence of the chr14:57152567-70490840 (hg19) locus of ten subjects (eight with thrombocytopenia and two controls from the family) according to Agilent’s recommendations. The genomic DNA was sequenced on a HISEQ 2000 sequencer (Illumina, San Diego, USA) as paired-end 75 bases, with an average depth of 100×. The bioinformatic analysis was performed using the NextGENe software (Softgenetics, PA, USA), which provides alignment, variant calling and filtering. Variant calling was performed with an allele frequency of >0.2 and a balanced ratio of forward and reverse strands of >0.1. In other words, a variant was called in two steps. First, it should be present in at least 20% of the sequence reads. Second, these reads should be present in both strands, with at least 10% of them being from either the forward or reverse strand.

### Plasmid construction, cell culture, transfection, and immunofluorescence

Total RNA was extracted and purified from HeLa cells with the Trizol reagent. The first strand cDNA synthesis was conducted using SuperScriptII Reverse Transcriptase with oligo(dT)_12-18_ (Invitrogen, Cergy Pontoise, France). The *ACTN1* cDNA sequence was amplified using forward primer 5'- ATGGACCATTATGATTCTCAGCAAACC -3' and reverse primer 5'- GAGGTCACTCTCGCCGTACAGC -3'. The PCR was performed with the Platinum® Taq DNA Polymerase High Fidelity kit using the conditions recommended by the supplier (Invitrogen, USA). The PCR products were purified using the QIAquick Gel Extraction Kit (Qiagen, Courtaboeuf, France) and cloned into pcDNA3.1/V5-His plasmid by TOPO® TA cloning (Invitrogen). The *ACTN1* p.Arg46Gln missense mutation was introduced into the plasmid construct by directed mutagenesis using the QuikChange^®^ Lightning Site-directed Mutagenesis Kit (Agilent, Massy, France). The mutated oligonucleotides corresponding to the p.Arg46Gln missense mutation were 5'- ACTCCCACCTCCAGAAGGCGGGGAC-3' and 5'- GTCCCCGCCTTCTGGAGGTGGGAGT-3' (the introduced nucleotide change is underlined).

COS-7 cells (ATCC^®^ CRL-1651™) were cultured in Dulbecco’s Modified Eagle’s medium supplemented with 10% fetal bovine serum. Wild-type (WT) and p.Arg46Gln *ACTN1* plasmid DNA (pDNA) constructions were transfected into COS-7 cells with the Lipofectamine 2000 reagent (Invitrogen). Then, 800 ng pDNA was mixed with Opti-MEM (Invitrogen) and Lipofectamine 2000 (Invitrogen) and incubated for 20 min at RT. After two washes with PBS 1×, the cells were transfected with the pDNA-Lipofectamine 2000 complex in serum-free medium for 8 h at 37°C, 5% CO_2_. The cells were washed with PBS 1× and cultured for 48 h in Dulbecco’s Modified Eagle’s medium supplemented with 10% fetal bovine serum. The cells were fixed in 100% methanol for 5 min at -20°C for the immunofluorescence analysis. After fixation and three 5-min washes with PBS 1×, the cells were permeabilized with 0.2% Triton X-100 in PBS for 15 min at RT. After 5-min washes with PBS 1×, nonspecific binding was prevented by blocking with 5% nonfat milk in PBS 1× (30 min, RT). The cells were then incubated with a FITC conjugated anti-V5 mouse monoclonal antibody in PBS 1×/5% nonfat milk (1:500 dilution, Life Technologies) for 1 h at RT. After three 5-min washes with PBS 1×, the cells were incubated for 30 min at RT with 165 nM Alexa Fluor^®^ 594 phalloidin (Life Technologies) in PBS 1×/5% nonfat milk for F-actin staining. Finally, after three 5-min washes with PBS, the slides were mounted with SlowFade® Gold Antifade Reagent with DAPI (Life Technologies) and examined under a Olympus BX-61 microscope. The images were collected with a ×60 objective lens.

**Figure 2:**
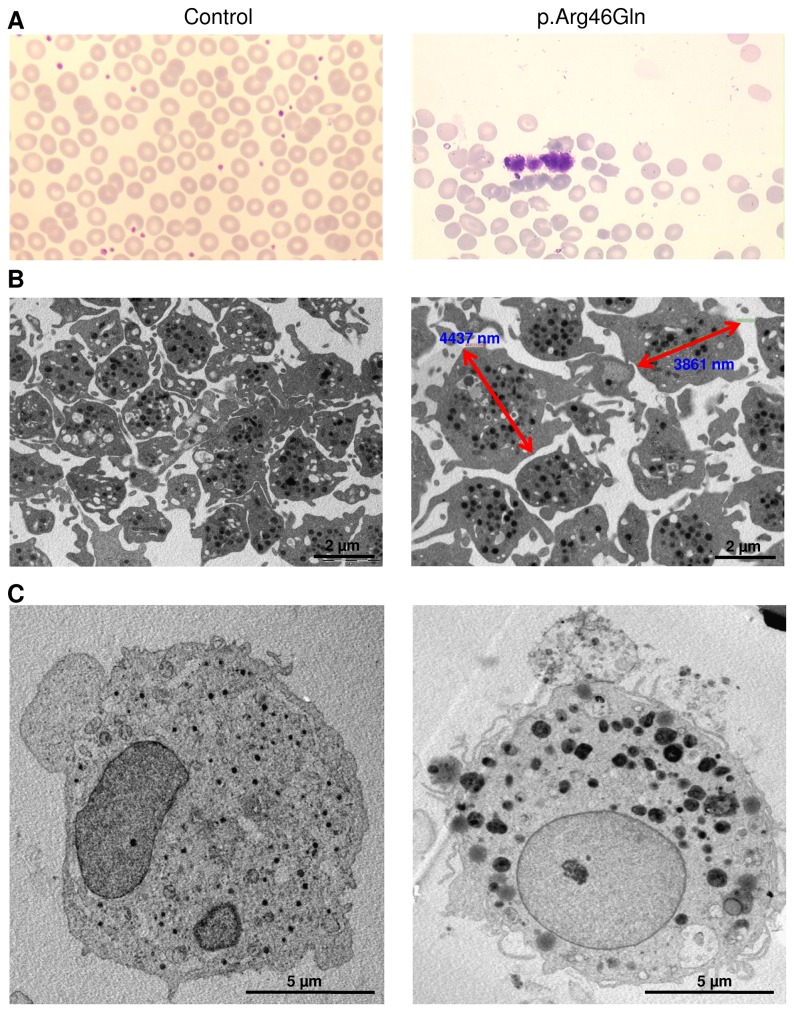
Morphology of platelet and MK cells from the proband and a control. (**A**) Morphology of platelets in the bone marrow smears, which were stained with May-Grunwald Giemsa. The platelet in the proband is much larger than its normal size by reference to the red blood cells and to the control. The analysis was performed on an optical microscope (Leica DMR) with a 50×/1.40 numerical aperture oil objective lens (Leica). (**B**) Morphology and size (indicated by red arrows in the proband) of platelets from the peripheral blood observed by electronic microscopy (magnification of 10000×). (**C**) Morphology of a representative cultured MK from the bone marrow observed by electronic microscopy (magnification of 6000×). Ultrathin sections were examined by a JEM-1010 transmission electron microscope (JEOL) at an accelerating voltage of 80 kV.

## Results

### The proband’s characteristics

The proband was subjected to bone marrow morphological examination by optical microscopy; whereas no abnormalities in the MK, erythroid and myeloid lineages were found (data not shown), the platelets were much larger than normal (Figure **2A**). Examination of her peripheral blood by optical microscopy revealed large platelets, too (data not shown). Under examination of electronic microscopy, her platelets in the peripheral blood were typically of around 4 µm in size (Figure **2B**), which is an approximately 60% increase as compared with normal controls. Further, after eleven days in culture, MK cells from the proband’s bone marrow were examined by electron microscopy. Compared with control MKs, the proband’s cells contained large granules with a heterogeneous distribution and aspect, and the cytoplasm was disorganized with fewer components. However, the demarcation membrane system [19] appeared to be preserved with many membrane invaginations (Figure **2C**).

She was analyzed using an *in vitro* platelet aggregation test, and no abnormalities in the platelet activation pathway were found. In addition, her hematopoietic progenitor cells obtained from bone marrow aspiration had a growth rate of 37 MK colonies, which fell within the normal range of between 30 and 140 colonies.

### Identification of a large macrothrombocytopenia pedigree with autosomal dominant inheritance

Starting from the aforementioned proband, we constructed a six-generation pedigree through extensive genealogical studies (Figure **1**). Of the 55 family members (all of whom live or lived in north Finistère, a region of 900,000 inhabitants in Brittany, in the north-western part of France) available for platelet count analysis, 26 had a platelet number lower than the normal value (>150 G/l) and had large platelets with anisocytosis in the peripheral blood. They were thus diagnosed with macrothrombocytopenia (the platelet number and the current age of each subject are summarized in Table **1**). The availability of the phenotypic data from this large number of subjects led to the establishment of a mode of autosomal dominant inheritance in this large pedigree (Figure **1**).

**Table 1:** Current age, platelet count, and genotype of the analyzed subjects in the pedigree.

Thrombocytopenia group				Non-thrombocytopenia group			
Subject number	Age (year)	Platelet count (G/l)	Genotype	Subject number	Age (year)	Platelet count (G/l)	Genotype
IV-02	69	97	p.Arg46Gln/WT	IV-01	66	314	WT/WT
IV-04	71	111	p.Arg46Gln/WT	IV-03	64	190	WT/WT
IV-06	75	98	p.Arg46Gln/WT	IV-05	72	228	WT/WT
IV-07	70	74	p.Arg46Gln/WT	IV-08	76	162	WT/WT
IV-09	88	112	p.Arg46Gln/WT	V-03	45	230	WT/WT
IV-10	83	63	p.Arg46Gln/WT	V-05	43	196	WT/WT
V-01	39	66	p.Arg46Gln/WT	V-06	44	219	WT/WT
V-02	44	57	p.Arg46Gln/WT	V-08	49	242	WT/WT
V-04	40	96	p.Arg46Gln/WT	V-09	46	242	WT/WT
V-07	44	63	p.Arg46Gln/WT	V-10	48	242	WT/WT
V-11	49	61	p.Arg46Gln/WT	V-12	52	217	WT/WT
V-14	53	98	p.Arg46Gln/WT	V-13	66	262	WT/WT
V-18	64	110	p.Arg46Gln/WT	V-15	52	277	WT/WT
V-19	45	91	p.Arg46Gln/WT	V-16	63	246	p.Arg46Gln/WT
V-21	56	97	p.Arg46Gln/WT	V-17	60	193	WT/WT
V-22	49	122	p.Arg46Gln/WT	V-20	70	246	WT/WT
VI-05	15	131	p.Arg46Gln/WT	V-23	48	208	WT/WT
VI-06	18	134	p.Arg46Gln/WT	VI-01	16	191	WT/WT
VI-07	20	125	p.Arg46Gln/WT	VI-02	13	210	WT/WT
VI-10	22	107	p.Arg46Gln/WT	VI-03	16	176	WT/WT
VI-14	15	133	p.Arg46Gln/WT	VI-04	20	270	WT/WT
VI-15	13	114	p.Arg46Gln/WT	VI-08	17	241	WT/WT
VI-16	36	56	p.Arg46Gln/WT	VI-09	22	217	WT/WT
VI-19	17	121	p.Arg46Gln/WT	VI-11	24	314	WT/WT
VI-20	16	126	p.Arg46Gln/WT	VI-12	41	311	WT/WT
VI-21	24	73	p.Arg46Gln/WT	VI-13	42	295	WT/WT
Mean ± SD		97.54 ± 25.78		VI-17	27	357	WT/WT
				VI-18	33	179	p.Arg46Gln/WT
				VI-22	21	204	WT/WT
				Mean ± SD		237.21 ± 47.52	

The mean platelet count in the 26 subjects with thrombocytopenia is 97.54 ± 25.78, which is significantly lower than that in the 29 healthy family members (237.21 ± 47.52, Table **1**). None of the subjects with thrombocytopenia showed either a bleeding diathesis or any other clinical features, such as neurological abnormalities, deafness, renal or ocular disease.

### Identification of the causative gene by positional cloning and confirmation by NGS

We had previously analyzed a non-syndromic thrombocytopenia candidate gene, *MASTL* [20], in this family without finding any causative mutations. Given that our patients did not have any clinical features that could provide clues as to the underlying causative genes, we decided to perform a genome-wide linkage analysis, an approach made feasible by the availability of a large number of thrombocytopenic (n = 26) and healthy (n = 29) subjects. Using multipoint parametric linkage analyses, we obtained a maximum LOD score of 7.6 for marker D14S1029 located on 14q22. The linked region extends over 10 Mb (Chr14:59,600,000–70,500,000 [hg19]). Of the 130 genes contained within this region (identified via the UCSC Genome Browser), only five (*ACTN1*, *DHRS7*, *HIF1A*, *KIAA0247* and *PPM1A*) are highly expressed in the blood and bone marrow. Of these, *ACTN1*, which encodes alpha-actinin 1, was regarded as the most likely candidate by virtue of its functional role in the cytoskeleton and by analogy to some of the known macrothrombocytopenia genes, such as *MYH9* [2,3], *FLNA* [4] and *TUBB1* [6]. The coding sequences plus immediate flanking intronic sequences of *ACTN1* (NM_001130004.1) were therefore analyzed first. Only one mutation was found to be co-segregated with the disease phenotype and was absent in 288 control individuals from our local population, as well as from the public dbSNP database and the 1000 Genomes Project dataset; it was a single-base substitution located within exon 2 of the *ACTN1* gene (c.137GA), which was predicted to result in a missense mutation, p.Arg46Gln. Two of the 29 healthy subjects, V-16 and VI-18, were also found to carry the mutation in the heterozygous state. [Average platelet volume in each subject is around 12 fL, the upper limit of the normal range (8-12 fL).] The penetrance of the missense mutation was thus 93% (26/28).

To ensure the absence of other clinically relevant mutations or variants in the locus segregating with the phenotype, we performed targeted NGS sequencing on eight affected (V-01, V-02, V-07, V-19, V-22, VI-07, VI-20 and VI-21) and two unaffected (IV-01 and V-09) individuals. Alignment to hg19 and NGS analyses were performed with the NextGENe software. Only one coding variant was found to co-segregate with the disease but also be absent in public databases (Table **2**); it was *ACTN1* p.Arg46Gln.

**Table 2:** Targeted sequencing data of the linkage-mapped locus by NGS.

Suject number^a^	Total variants	Coding variants^b^	Not reported coding variants^c^
V-01	95065	880	688
V-02	102725	1060	847
V-07	85302	1117	887
V-19	97894	1085	868
V-22	83526	898	736
VI-07	85817	1104	868
VI-20	167675	959	774
VI-21	65709	1019	799
IV-01	199490	1003	816
V-09	93563	920	751
Comparison^d^	429	8	1

a The first eight are subjects with thrombocytopenia and the last two are family controls.

^b^ Including variants in their immediate flanking sequence [-12; +12]

c Variants not reported in public databases (dbSNP and 1000 genomes).

d Variants shared by thrombocytopenic subjects but absent in family controls.

### Functional analysis of the *ACTN1* p.Arg46Gln missense mutation

**Figure 3:**
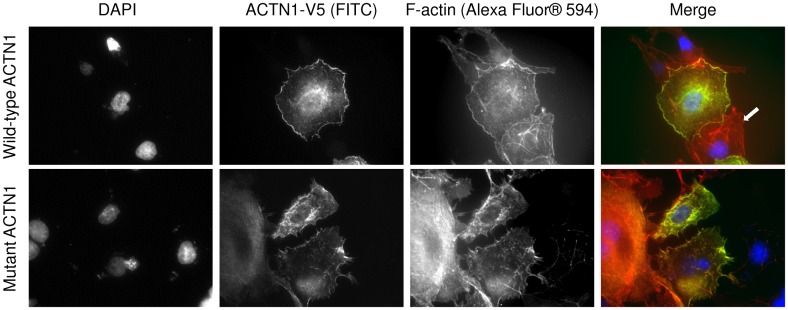
Evaluation of the functional effect of *ACTN1* p.Arg46Gln on transfected COS-7 cells by immunofluorescence. COS-7 cells transfected with either wild-type (top panel) or mutant (lower panel) *ACTN1* plasmid construction (as well as non-transfected cells) were stained with three different flurochromes as indicated in the Figure. In the merged image, overexpressed α1-actinin, normal F-actin network and counterstained nuclei appear in green, red and blue, respectively. In the top right panel, the arrow indicates an untransfected cell. Fluorescence was visualized on an Olympus BX-61 fluorescence microscope with a 60×/1.40 numerical aperture oil objective lens (Plan Apo, Olympus). Image capture was performed with a Diagnostics Instruments CCD camera system using SPOT Advanced software version 3.2.4 (Diagnostic Instruments, Sterling Heights, MI). Image processing was made with Image G (1.45S, NIH, USA).

COS-7 cells transfected with the WT *ACTN1* plasmid construct showed a fine and homogenous distribution of actin filaments in the cytoplasm, and WT ACTN1 co-localized with the actin filaments. In contrast, COS-7 cells transfected with the mutant plasmid construct showed discrete anomalies of the actin network and less fine ACTN1 distribution in the cytoplasm (Figure **3**).

## Discussion

In this study, we successfully identified a missense mutation in the *ACTN1* gene (p.Arg46Gln) as the cause of an autosomal dominant form of isolated macrothrombocytopenia in a large, six-generation Caucasian pedigree (Figure **1**). This was achieved by a positional cloning strategy followed by targeted NGS sequencing of the 13 Mb locus mapped to chromosome 14q22. Six missense mutations in the *ACTN1* gene, including p.Arg46Gln, have been recently described in six small Japanese families with macrothrombocytopenia using whole-exome sequencing combined with Sanger sequencing [21]. These independent findings from two distinct populations (Caucasian and Asian) strongly support the involvement of the *ACTN1* gene in macrothrombocytopenia.

Alpha-actinins (ACTNs) are composed of four isoforms encoded by four distinct loci. Isoforms 2 and 3 are expressed in the sarcomere of muscle tissue, whilst ACTN1 and ACTN4, the non-muscle isoforms, are widely distributed [22]. ACTN isoforms are organized as anti-parallel dimers with an actin-binding domain at the N terminus, through which they crosslink actin filaments into bundles [23]. The *ACTN1* p.Arg46Gln missense mutation affected an evolutionarily conserved amino acid and occurred within the functionally critical actin-binding domain (ABD) of the protein. We analyzed the functional effect of this mutation in transfected COS-7 cells by immunofluorescence and found that it caused discrete disorganization of the actin and alpha-actinin 1 filaments (Figure **3**). This observation concurs with that obtained from the functional analysis of the same missense mutation in a different cell line (CHO) [21]. In particular, we also provided data on the morphology of bone marrow-derived MKs, the platelet progenitors from a patient. In the electron microscopy examination, MK cells from the proband showed abnormal cytoplasm organization characterized by heterogeneous giant granules (Figure **2C**). This morphological change is similar to that observed in cells with a mutation in another actin partner, filamin A [4]. Numerous invaginations of the membrane can be observed, suggesting that proplatelet formation is active.

Due to the large number of the subjects studied in our pedigree, we were able to establish a clear model of autosomal dominant inheritance of the *ACTN1*-associated macrothrombocytopenia and demonstrated a high penetrance of the *ACTN1* p.Arg46Gln mutation. Interestingly, two family members without thrombocytopenia in our pedigree carried the *ACTN1* p.Arg46Gln missense mutation. It did not escape our attention that they were first degree relatives, one being the mother (V-16), the other the son (VI-18) (Figure **1**). It is possible that they both harbor an unidentified genetic factor that counteracted the effect of the *ACTN1* mutation.

Another important point is that the single-base substitution (c.137GA) resulting in the recurrent p.Arg46Gln missense mutation is a CpG mutation, consistent with the notion that CpG dinucleotide site is the most common mutational hotspot in the human genome [24]. As a matter of fact, our survey of the other five thrombocytopenia-causing *ACTN1* missense mutations [21] revealed that, apart from p.Gln32Lys, all the other mutations are also CpG mutations, too. This observation implies that *ACTN1* mutations are almost certainly present in other populations and probably account for some unidentified patients with mild to moderate isolated macrothrombocytopenia. Finally, the discovery of causative *ACTN1* mutations in subjects with isolated thrombocytopenia shows again the pivotal importance of genetic findings in disease diagnosis and treatment. In this regard, we would like to reiterate that the proband in our pedigree was initially diagnosed with immune thrombocytopenia and consequently received inappropriate and clearly harmful splenectomy.

In summary, using positional cloning and targeted NGS, we identified a causative mutation in the *ACTN1* gene in a large French family with autosomal dominant macrothrombocytopenia. Our study concurred with the recently published Japanese study [21], adding *ACTN1* to the growing list of thrombocytopenia genes [1].
